# Supplementary figures and images for: Rsp5/NEDD4 and ESCRT regulate TDP-43 toxicity and turnover via an endolysosomal clearance mechanism
Source: J Cell Biol. 2026 Jan 7;225(2):e202212064. doi: 10.1083/jcb.202212064 (PMC12777955; doi:10.1083/jcb.202212064)

Figure 1D

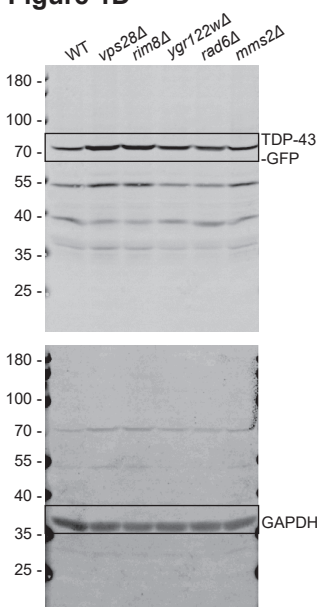

Supplement: SourceData F1 — is the source file for Fig. 1. [file jcb_202212064_sourcedataf1.pdf]

Figure 2A

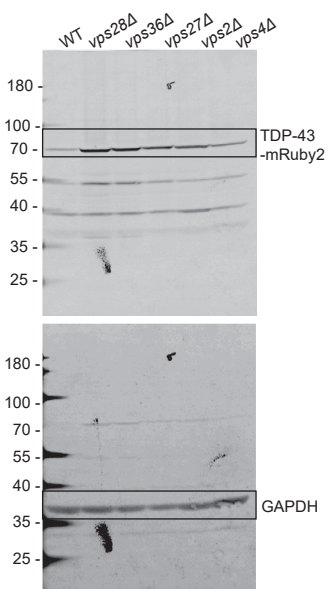

Figure 2C

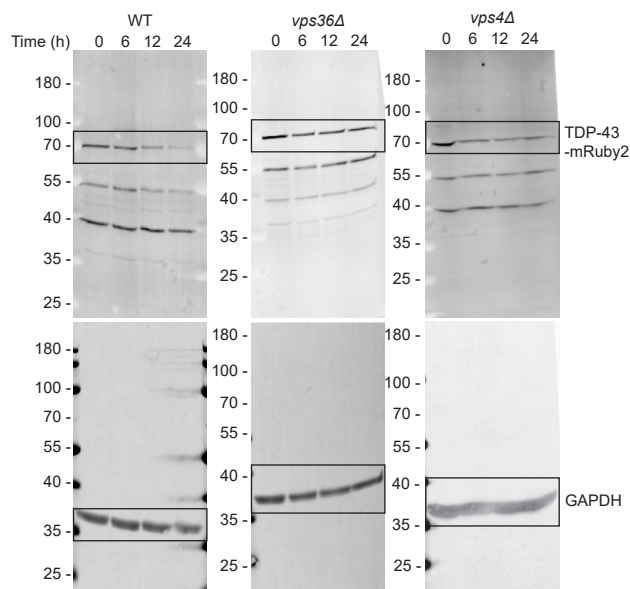

Supplement: SourceData F2 — is the source file for Fig. 2. [file jcb_202212064_sourcedataf2.pdf]

**Figure 3C**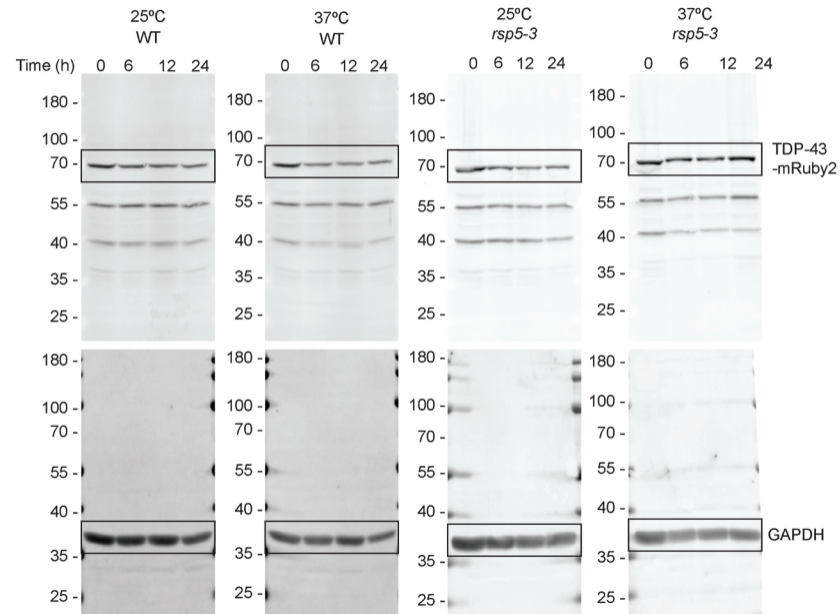**Figure 3F**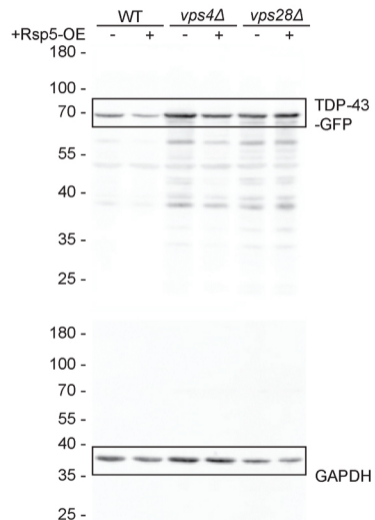

Supplement: SourceData F3 — is the source file for Fig. 3. [file jcb_202212064_sourcedataf3.pdf]

Figure 4A

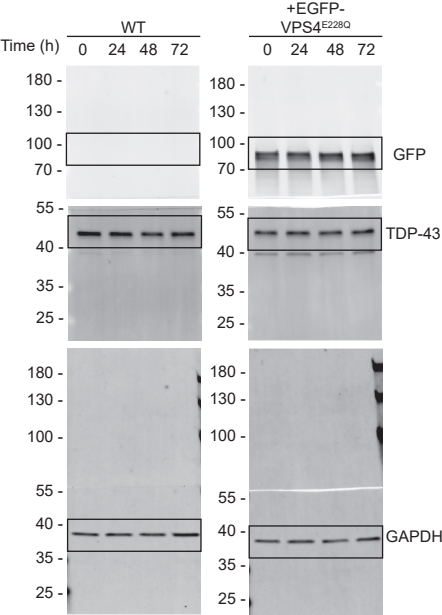

Supplement: SourceData F4 — is the source file for Fig. 4. [file jcb_202212064_sourcedataf4.pdf]

**Figure 5A**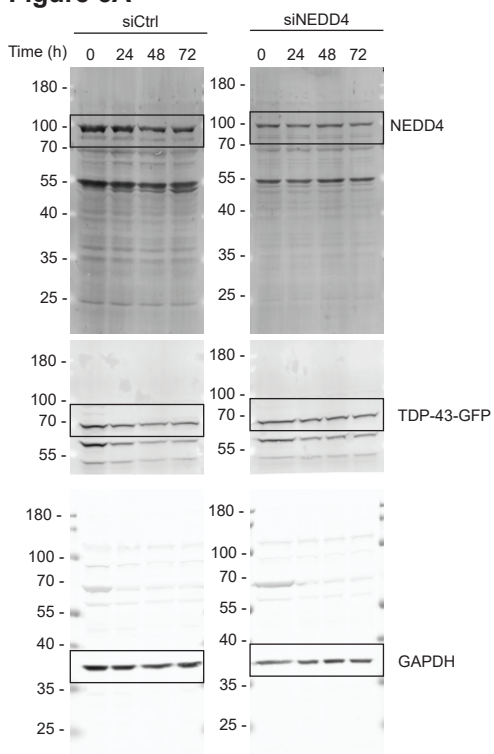**Figure 5B**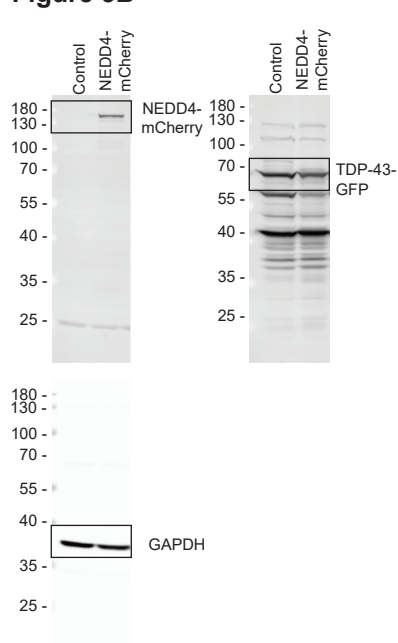**Figure 5E**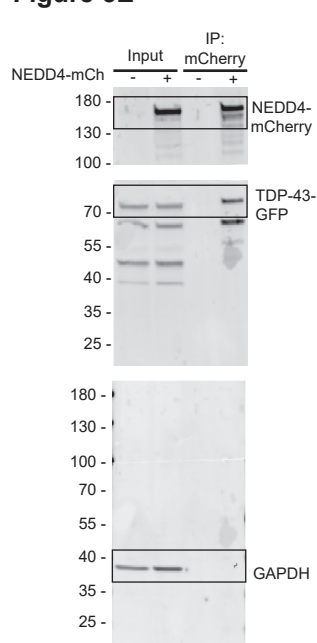**Figure 5F**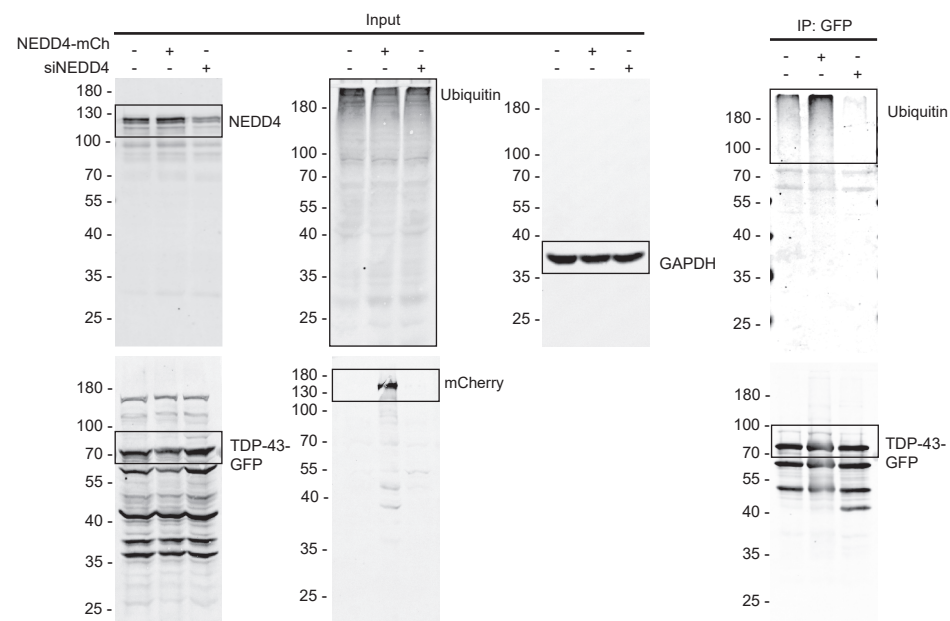

Supplement: SourceData F5 — is the source file for Fig. 5. [file jcb_202212064_sourcedataf5.pdf]

Figure S2A

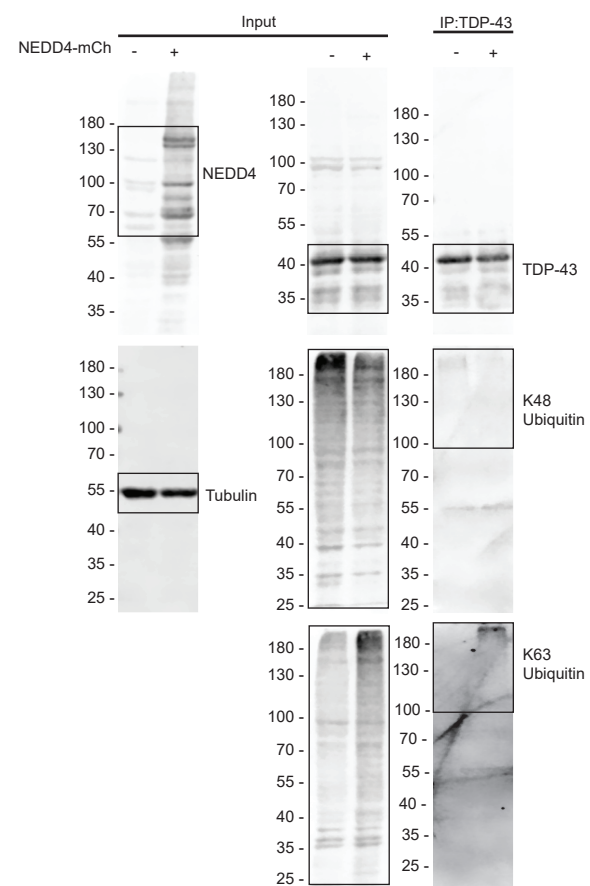

Figure S2B

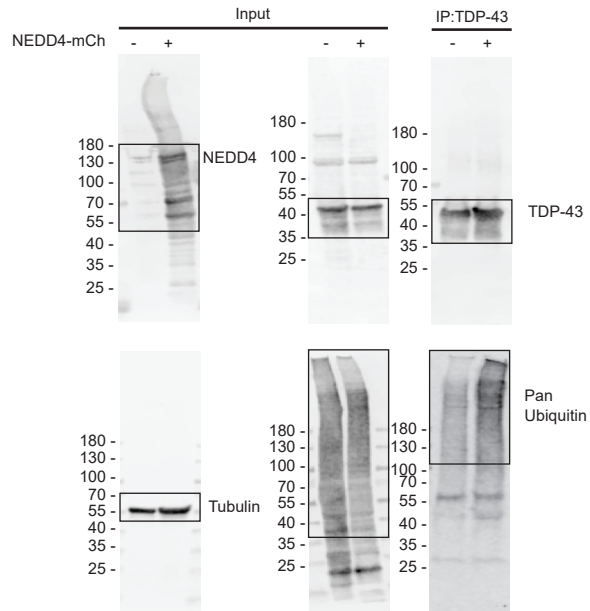

Supplement: SourceData FS2 — is the source file for Fig. S2. [file jcb_202212064_sourcedatafs2.pdf]

Figure S3

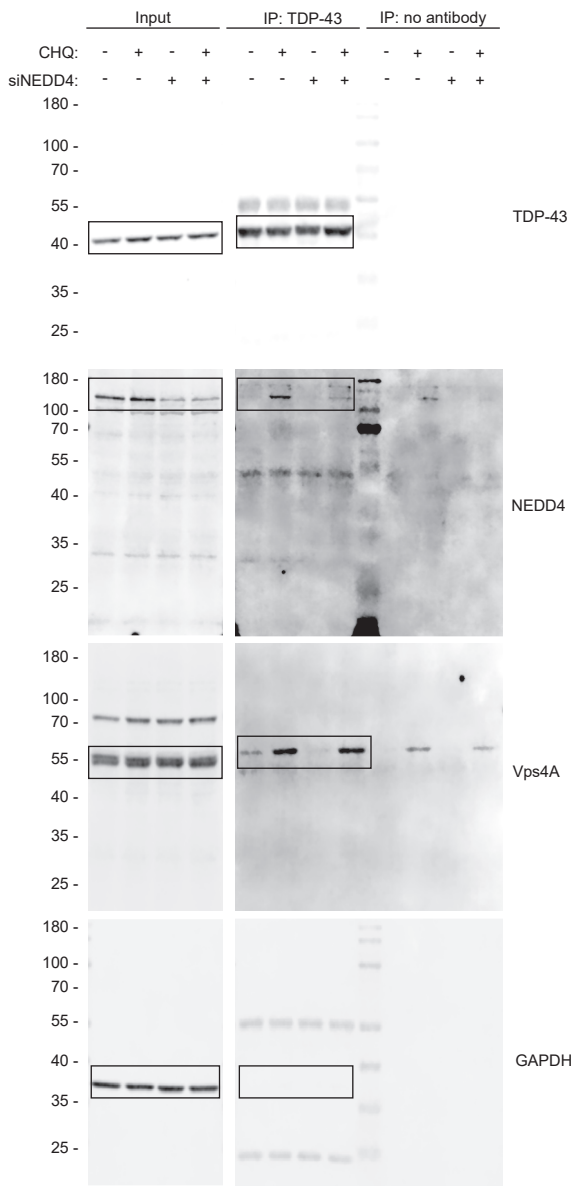

Supplement: SourceData FS3 — is the source file for Fig. S3. [file jcb_202212064_sourcedatafs3.pdf]
